# 10 years of CRISPR/CAS genomic engineering in *Yarrowia lipolytica*

**DOI:** 10.1007/s00449-026-03347-1

**Published:** 2026-05-23

**Authors:** Rodrigo Gonçalves Dias, Fernanda Pinheiro Moreira Freitas, Samuel Lessa Barbosa, João Victor Marques Gonçalves Assis, Thaynara Lorenzoni Entringer, Juliana Silva Carneiro Fonseca, Miguel Edmundo Romanizio, Bruno Brayan Zanotti Pimentel, Maria Emilene Martino Campos-Galvão, Nívea Moreira Vieira, Luciano Gomes Fietto, Agustin Zsögön, Wendel Batista da Silveira

**Affiliations:** 1 Department of Microbiology, Universidade Federal de Viçosa, Viçosa, MG 36570-900 Brazil; 2 Institute of Biotechnology Applied to Agriculture (BIOAGRO), Universidade Federal de Viçosa, Viçosa, MG 36570-900 Brazil; 3 Department of Biochemistry and Molecular Biology, Universidade Federal de Viçosa, Viçosa, MG 36570-900 Brazil; 4 Department of Plant Biology, National Institute of Science and Technology on Plant Physiology Under Stress Conditions, Universidade Federal de Viçosa, Viçosa, MG 36570-900 Brazil

**Keywords:** Bioinformatics tools, Metabolic engineering, Molecular biology, Oleaginous yeasts.

## Abstract

*Yarrowia lipolytica* is a versatile cell factory widely used in bioprocesses for producing lipids, organic acids, and other high-value compounds. Historically, its genetic engineering was constrained by low homologous recombination (HR) efficiency and the predominance of non-homologous end joining (NHEJ), limiting strain development and metabolic pathway optimization. The advent of CRISPR-Cas technologies has revolutionized genome editing in *Y. lipolytica*, enabling precise, efficient, and multiplexed modifications. Innovations such as pCAS1yl and pCRISPRyl plasmids, along with genomic Cas9 integration, have enhanced targeted editing efficiency. CRISPR applications now extend to metabolic engineering for lipids, itaconic acid, erythritol, and other compounds. Beyond canonical Cas9, alternative systems, including CRISPRa, Cas12a, base editors, and sgRNA libraries, provide increased flexibility and functional diversity. Central to these approaches is the rational design of guide RNAs (gRNAs), supported by bioinformatics platforms such as CHOPCHOP v3, CRISPOR, CCTop, and Cas-OFFinder, which assist in target site selection, off-target prediction, and editing optimization. This review summarizes the main CRISPR/Cas9 applications in *Y. lipolytica*, highlighting key engineered strains and emphasizing the critical role of bioinformatics in improving editing strategies. We also propose a pipeline for systematic gRNA design based on published evidence and discuss future perspectives, including the integration of machine learning, artificial intelligence, and emerging CRISPR variants to further advance yeast metabolic engineering.

**Graphical abstract:**

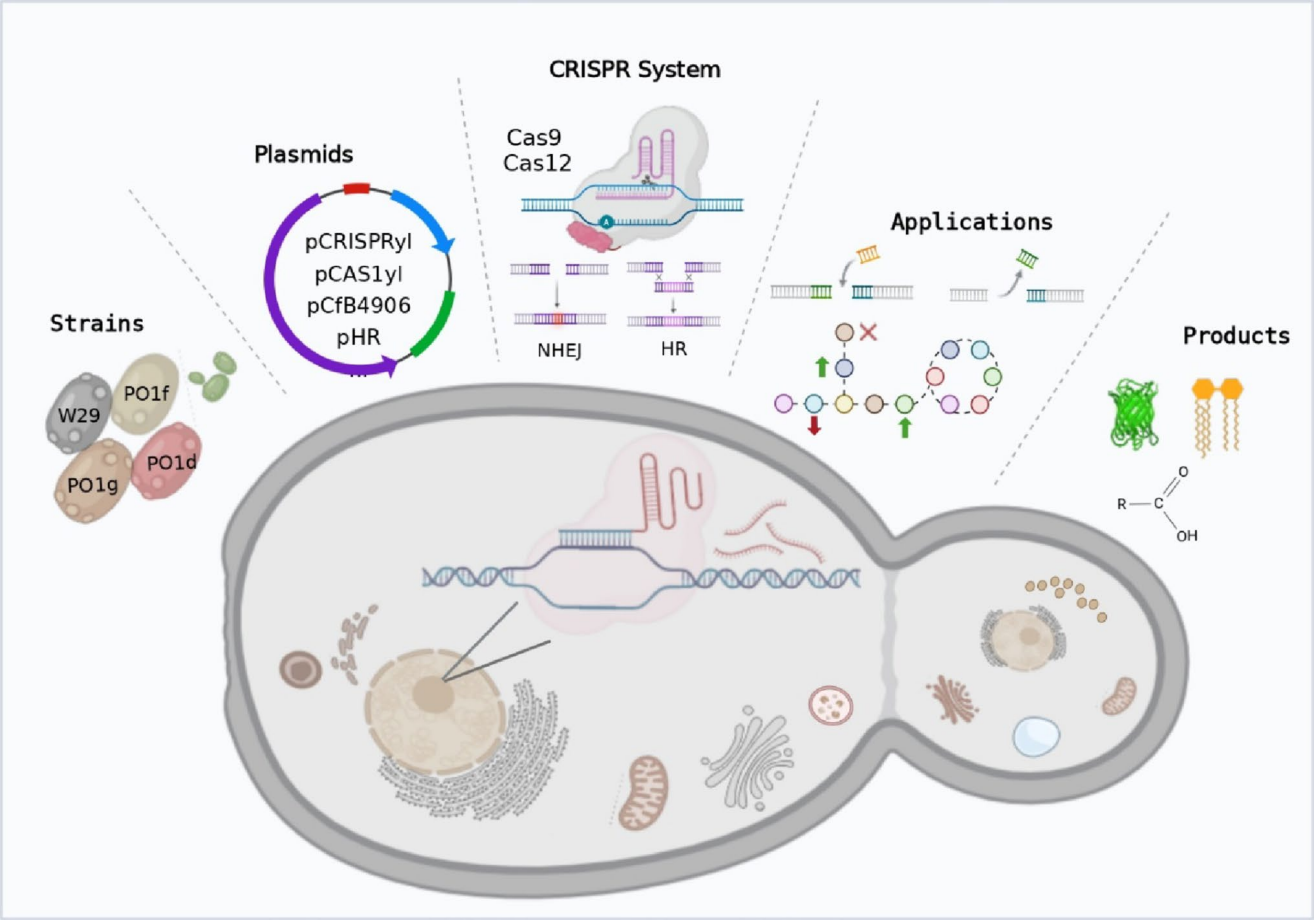

**Supplementary Information:**

The online version contains supplementary material available at 10.1007/s00449-026-03347-1.

## Introduction

The intensifying global demand for environmentally sustainable and economically competitive bioprocesses for the synthesis of high-value molecules has become a primary driver of innovation in microbial biotechnology. This transition toward a bio-based economy has intensified the search for robust and genetically tractable microbial chassis capable of supporting scalable and programmable biomanufacturing. Yeasts have emerged as powerful cellular factories for the synthesis of metabolites, enzymes, and other biomolecules with applications in food, energy, and health sectors. While the conventional yeast *Saccharomyces cerevisiae* has long dominated industrial fermentation, non-conventional species have gained prominence due to their metabolic diversity and robustness under harsh conditions [1, 2].

Among non-conventional yeasts, *Yarrowia lipolytica* stands out as an oleaginous species recognized as Generally Regarded as Safe (GRAS) and with remarkable metabolic versatility. This yeast, which is considered the oleaginous yeast model, can use hydrophobic substrates such as fatty acids and oils, secrete high levels of proteins, and accumulate significant quantities of lipids. Its ability to produce organic acids, carotenoids, and polyols, including erythritol, has made it a valuable platform for both academic and industrial applications, and can be rationally leveraged through metabolism engineering [3–5]. However, metabolic versatility alone does not ensure industrial performance. The capacity to rationally and predictably reprogram cellular networks remains the decisive factor determining whether *Y. lipolytica* can transition from a promising organism to a fully optimized industrial chassis. For many years, this was hindered by the biological predominance of non-homologous end joining (NHEJ) over homologous recombination (HR), rendering precise targeted integration inefficient. Consequently, strain construction necessitated laborious iterative cycles and marker recycling to accumulate multiple modifications, significantly extending engineering timelines [6].

The advent of CRISPR/Cas systems marked a conceptual and technological turning point in genome engineering. As an RNA-guided endonuclease, Cas9 enables the generation of double-strand breaks at specific genomic locations which, when combined with donor DNAs containing homology arms, significantly increase the frequency of targeted modifications. This transition toward precise and scalable genome editing has been accompanied by several host-specific adaptations for *Y. lipolytica*, including the development of co-expression plasmids such as pCAS1yl and pCRISPRyl, genomic integration of the nuclease, and the expansion of the CRISPR toolkit with variants such as Cas12a and dCas9 [7–9]. In this context, the field has progressed beyond simply demonstrating the functionality of CRISPR in this yeast; the central question has shifted toward understanding to what extent this technology has reshaped strain engineering strategies, expanded the design space for metabolic rewiring, and reduced the gap between laboratory-scale modifications and industrial implementation. Recent advances reflect a clear trend: the establishment of CRISPR as a standard tool for engineering *Y. lipolytica*. Beyond single-gene manipulations, CRISPR now supports library construction, large-scale gene regulation, and multiplex genome editing. The combination with rapid DNA assembly methods such as Golden Gate, together with the adoption of Cas variants including Cas12a and dCas9, promises greater flexibility, specificity, and efficiency. In parallel, the rational design of CRISPR/Cas components, particularly guide RNAs (gRNAs), has become a critical step in genome engineering workflows. Several bioinformatics platforms, such as CHOPCHOP v3, CRISPOR, CCTop, and Cas-OFFinder, support target selection, off-target prediction, and editing optimization. More recently, machine learning–based tools have further improved the accuracy of gRNA prediction. Among them, DeepGuide was specifically developed for *Yarrowia lipolytica*, using deep learning models trained on genome-wide CRISPR screening datasets to predict sgRNA activity with higher accuracy in this species. By incorporating organism-specific features, DeepGuide provides a more reliable framework for designing efficient guides and highlights the growing importance of computational approaches in optimizing genome editing strategies [7, 8, 10–12].

In this review, we examine the key milestones achieved over the past decade of CRISPR application in *Yarrowia lipolytica*. We highlight the major studies that have provided foundational insights for the development and optimization of CRISPR-based platforms in this yeast. In addition, we summarize the main applications of CRISPR technologies in *Y. lipolytica*, including their use in metabolic engineering and strain improvement for the production of value-added compounds. We also discuss the existing gap between scientific publications, patent developments, and the industrial implementation of robust engineered strains. Furthermore, we provide an overview of the main bioinformatics tools used for the rational design of CRISPR systems, including platforms widely applied for guide RNA design as well as tools specifically developed for *Y. lipolytica*. Finally, we discuss current limitations and outline future perspectives, highlighting emerging strategies, such as advanced CRISPR variants, machine learning–assisted design, and integrative genome engineering approaches, that may further accelerate the development of robust and predictable *Y. lipolytica* cell factories for industrial biotechnology.

### A chronology of the development of CRISPR/Cas platforms

**Fig. 1:**
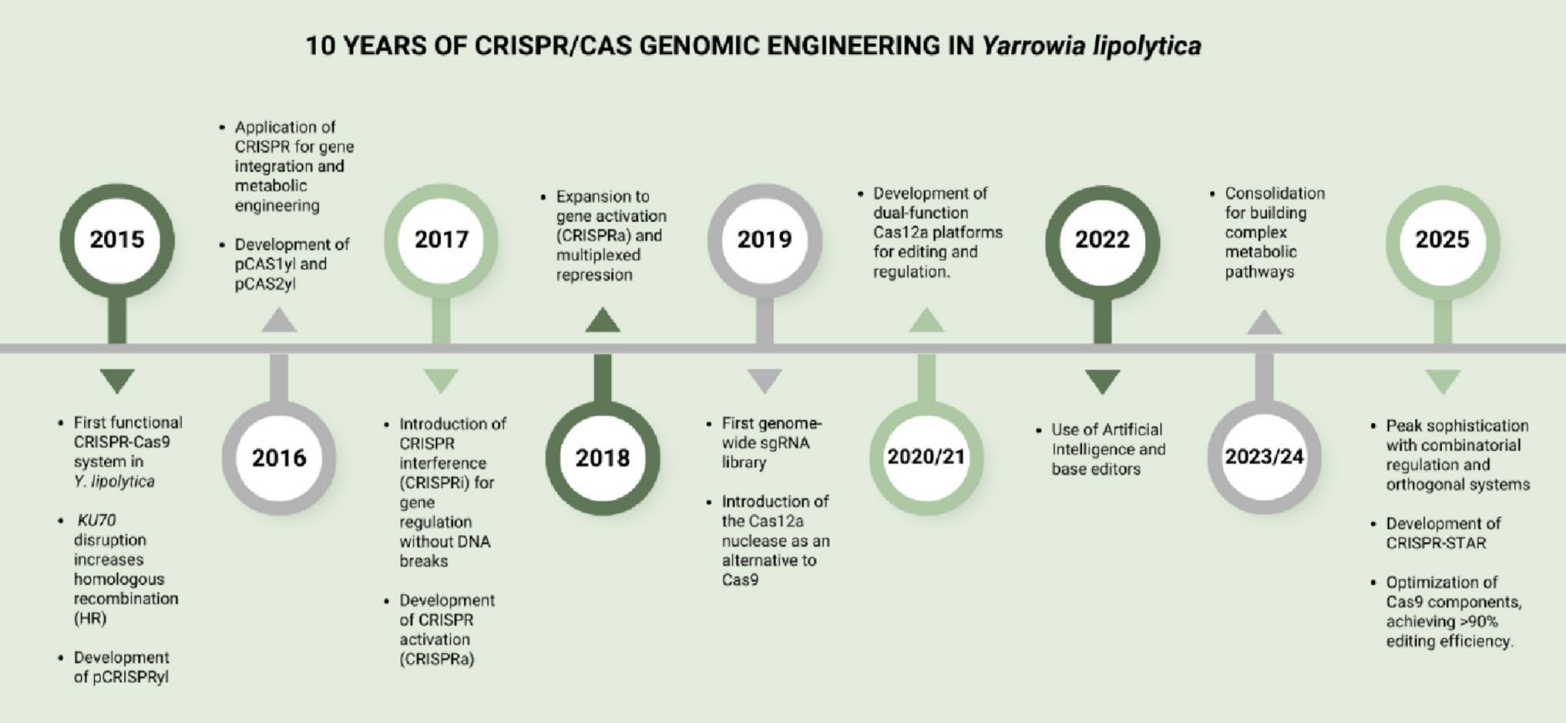
10 years of CRISPR/Cas genomic engineering in *Y. lipolytica*.

Over the past decade, the oleaginous yeast *Y. lipolytica* has evolved from a promising non-conventional host into a mature chassis for sustainable biomanufacturing (Fig. 1) [1, 2]. This transition was primarily enabled by the rapid adoption and refinement of CRISPR/Cas technologies tailored to *Y. lipolytica*’s physiology and DNA repair idiosyncrasies [7]. Together, these advances allowed the implementation of design-build-test-learn cycles and paved the way for improving the production of lipids, terpenoids, and specialty chemicals [13, 14]. The year 2015 marked the initial effort to establish a functional CRISPR/Cas9 system in *Y. lipolytica*. Furthermore, this foundational work demonstrated that disrupting *KU70*, a key gene in the NHEJ pathway, remarkably increased the frequency of homologous recombination (HR) [14]. This pivotal finding laid the conceptual groundwork for all future strategies to optimize genomic editing in *Y. lipolytica* by rebalancing its DNA repair bias. The practical output was the development of the modular plasmid pCRISPRyl, the first functional CRISPR toolkit for this yeast, combining optimized promoters for both Cas9 and sgRNA [9]. Building on the 2015 breakthrough, the CRISPR system was rapidly applied for broader metabolic engineering and genomic integration in 2016. It was first used to elucidate the xylose metabolism pathway, which is relevant for lignocellulosic biomass utilization [15]. Concurrently, researchers developed the first strategy for targeted gene integration via homologous recombination, identifying stable chromosomal loci (*AXP*, *XPR2*, *A08*, *D17*, *MFE1*) as ideal “landing pads” for biosynthetic pathways [9]. New systems, pCAS1yl and pCAS2yl, were introduced, incorporating hammerhead (HH) and hepatitis delta virus (HDV) ribozymes for autonomous sgRNA processing. This design allowed for multiple gene deletions [16]. This enabled the construction of specific integration vectors and led to the first functional assembly of a heterologous metabolic pathway in *Y. lipolytica*, aiming at lycopene production [17, 18].

*Y. lipolytica* uses NHEJ preferentially to repair double-strand breaks; therefore, strains that do not have the genes required for NHEJ (e.g. KU70, KU80 and DNL4) need homology regions larger than 500 bp to perform homologous recombination (HR) efficiently [9]. As a result, the insertion of genes of industrial interest by CRISPR depends on strains very sensitive to mutation, as their main repair mechanism has been deleted. This was the main drawback in developing robust industrial strains by CRISPR insertion. To circumvent this problem, interference systems were generated and tested in *Y. lipolytica*. A paradigm shift occurred in 2017 with the introduction of CRISPR interference (CRISPRi). This system used a catalytically inactive “dead” Cas9 (dCas9) to bind target DNA and modulate the transcription without creating double-strand breaks, allowing for reversible regulation of essential genes without compromising cell viability [18–20]. Using pCRISPRyl to repress multiple genes in the NHEJ pathway, it was demonstrated that repressing KU70, KU80, and DNL4 significantly increases HDR efficiency. The fusion of dCas9 to the repressor domain Mxi1 significantly enhanced silencing, from 38% to 87%. This established an elegant “edit the editor” strategy: using transient CRISPRi to knock down the NHEJ machinery, thereby “preparing” the cell for highly efficient subsequent editing with active CRISPR/Cas9 and a donor DNA template [18]. The development of a CRISPR activation (CRISPRa) system in a conceptual similar approach (using a dCas9 fused to the transcription activator VPR) enabled yeast growth on cellobiose through the activation of native β-glucosidase genes. This demonstrated CRISPR’s potential for rewiring native metabolic pathways [19].

The year 2019 was a watershed moment with the move to genomic-scale engineering. Researchers developed the first genome-wide sgRNA library for *Y. lipolytica*, covering 7,854 protein-coding genes with six guides per gene. The study introduced a dual-parameter analysis to evaluate guide performance: the Fitness Score (FS), which measures the impact of gene disruption on cell growth, and the new parameter Cutting Score (CS), which indicates the intrinsic efficiency of the sgRNA to induce a cut. This allowed us to distinguish between essential genes (low FS) and inefficient guides (high CS). The library revealed that over 94% of genes had at least one highly functional guide and identified trends of lower efficiency in telomeric and nucleosome-rich regions. This resource became a cornerstone for robust, high-throughput functional genomics and host engineering and developed a new library for disrupting almost every gene in the yeast [21]. In this period there was the diversification of the CRISPR toolbox with the introduction of Cas12a (Cpf1). Unlike Cas9, Cas12a recognizes distinct T-rich PAM sites and can autonomously process a single transcript into multiple crRNAs, simplifying the assembly of compact multiplex systems to edit multiple loci simultaneously. In addition, Cas12a also allows multiple alterations in the same gene because it cuts downstream of the PAM site, generating cohesive ends. Implementations in *Y. lipolytica* have demonstrated accurate and efficient multiplex genome editing. A significant advance was the engineering of a dual-purpose Cas12a platform capable of simultaneously disrupting genes and regulating transcription. This is possible using crRNAs of different sizes; 16 nt crRNAs can recognize the PAM site but cannot generate the structure necessary for the enzyme’s nuclease function [22]. In 2019, a Target-AID system that uses Cas9, cytidine deaminase, and an Uracil DNA Glycosylase Inhibitor was also established to make point modifications in the nucleotide sequence. Parallel improvements in vector architecture and polycistronic guide expression cassettes have made CRISPR editing more predictable and standardized across laboratories [23]. Until this year, efforts focused on generating industrially interesting strains that used CRISPR only to disrupt target genes, while the insertion of new genes was still performed using classical systems. CRISPR has also accelerated pathway-scale construction. Beyond Cas9/Cas12a editing, *Y. lipolytica* now benefits from repeated, markerless integration via refined Cre/lox workflows and from homology-independent targeted integration tools, both of which streamline stable assembly of extensive or modular pathways [23]. Together with portable vector sets for diverse wild-type strains, these methods have made a multi-round genome writing routine [24]. The technological arc translated into tangible bioproduction gains. In terpenoids, iterative genome editing and regulation delivered record sesquiterpene titers such as β-farnesene, including processes starting from waste lipids or lignocellulosic sugars, and broader progress has been documented across sclareol, β-ionone and other products [13, 25]. Overall, *Y. lipolytica*’s strengths in acetyl-CoA and NADPH supply, P450 expression, and ω-oxidation chemistry position it as a leading chassis for fatty acid derived chemicals and nutraceuticals [1].

In 2025, several landmark studies extended the CRISPR toolkit in *Y. lipolytica*. First, Chen et al. [26] developed CRISPR-STAR, a scaffold-RNA platform enabling simultaneous transcriptional activation and repression, achieving up to 55% improvements in fatty alcohol titers by co-activating FAR and repressing PEX10. Li et al. [27] harnessed random cytosine base editing to identify previously unknown genetic targets enhancing terpenoid biosynthesis. Parallel optimization of Cas9 components, including sgRNA promoters, Cas9 variants, and transient NHEJ suppression via KU70, pushed gene disruption efficiencies above 90%, while Zhang et al. [28] combined Cas12a with an orthogonal T7 system to enable multiplexed editing and transcript knockdown. Together, these studies illustrate that by 2025, CRISPR technologies in *Y. lipolytica* had matured into integrated platforms for editing, regulation, and large-scale discovery. Looking forward, base-editing toolkits will likely combine with genome-wide libraries and AI-assisted guide design (e.g., DeepGuide) to support high-throughput genotype-to-phenotype mapping, while prime editing, though powerful in other systems, remains to be established in *Y. lipolytica*. Emerging circuits combining CRISPRa and CRISPRi in one strain (e.g., CRISPR-STAR–style simultaneous activation and repression) hint at dynamic, closed-loop control over flux and burden. As these layers converge, we anticipate faster route-to-function for sustainable fuels, materials, and fine chemicals in this uniquely tractable oleaginous yeast [13, 14, 26].

As these tools matured, research increasingly focused on engineering strains capable of producing high-value compounds. At the same time, the growing number of patents involving CRISPR/Cas-modified *Y. lipolytica* reflects the effort to translate scientific advances into technological innovation.

### Applications of CRISPR/Cas-engineered ***Y***. ***lipolytica***

In the last decade, *Y. lipolytica* stood out as one of the most promising chassis for industrial biotechnology, mainly due to incorporation of CRISPR/Cas tools in metabolic engineering approaches [2]. In laboratory research, these genome editing systems have enabled precise gene deletions, pathway optimization, and heterologous gene integration, facilitating proof-of-concept studies aimed at improving the production of lipids, organic acids, and other value-added compounds [29, 30]. Such studies typically represent the first stage in the development pipeline, where genetic tools and engineered metabolic routes are validated at laboratory scale before being considered for technological development and industrial implementation [31].

At the laboratory level, CRISPR/Cas technology has been widely applied to develop optimized *Y. lipolytica* strains capable of producing high-value compounds, including carotenoids and terpenoids such as β-carotene [17, 24], zeaxanthin [24], β-ionone [24], β-farnesene [32] and longifolene [33], as well as aromatic and phenolic compounds such as resveratrol [34], polydatin [35], naringenin [7], dihydroquercetin [36], homogentisic acid [37], and 2-phenylethanol [38]. In addition, increasing lipid accumulation and the production of industrially relevant fatty acids, such as ricinoleic acid [39], arachidonic acid [40], and docosahexaenoic acid (DHA) [41], has also been a major objective. Metabolic pathways have also been engineered to optimize the production of industrial metabolites, including erythritol [14] and itaconic acid [42]. Detailed information on CRISPR/Cas-based genome engineering approaches in *Y. lipolytica*, including plasmid systems, delivery strategies, repair mechanisms, and targeted metabolic outcomes, is summarized in Online Resource 1.

The transition from laboratory-scale discoveries to industrial deployment generally follows a structured innovation pathway. Academic research typically generates proof-of-concept demonstrations of genome editing strategies and metabolic pathways, which may subsequently be protected through intellectual property mechanisms such as patents [43]. This protection plays a central role in technology transfer, enabling universities and research institutions to license innovations to companies or support the creation of startups aimed at commercial development [44]. In this context, patents act as an intermediate step connecting scientific advances to industrial implementation, facilitating investment and technological maturation until the process reaches pilot and commercial scales [45]. Consequently, the increasing number of patents involving CRISPR/Cas-engineered *Y. lipolytica* reflects not only scientific progress but also the growing interest in translating these advances into marketable biotechnological solutions.

A search conducted in the Espacenet database between 2015 and 2025, using the terms “*Yarrowia lipolytica*” and “CRISPR/Cas,” yielded 444 patent records, evidencing a consistent growth in the protection of innovations related to this yeast. This search not only reflects the economic relevance of the species but also reveals how the transition from academic advances to industrial applications is frequently mediated by intellectual property. Among the most notable applications are patents directed at the production of nutraceutical carotenoids, such as astaxanthin. Patent CN118272421B [46] describes the use of CRISPR/Cas9 to integrate the CrtW and CrtZ genes into strategic loci of the *Y. lipolytica* genome, resulting in strains capable of accumulating this metabolite. Similarly, patent WO2025103054A1 [47] presents a modified strain designed to produce different carotenoids (astaxanthin, canthaxanthin, and zeaxanthin) using xylose-inducible regulatory systems. Beyond pigments, the versatility of the yeast is also reflected in patents such as CN116179382A [48], which describes a strain with higher erythritol yield, achieved through the overexpression of genes from the xylose pathway and deletion of a dehydrogenase. These examples highlight the application of CRISPR/Cas both for introducing heterologous metabolic pathways and for optimizing native routes, focusing on high-value molecules. Despite the significant progress in genetic engineering of *Y. lipolytica* using CRISPR/Cas, the patent landscape still presents challenges. Many claims have considerable breadth, as in US20200165637A1 [49], which describes a CRISPR/Cas system applied to lipolytic yeasts, covering specific genes and integration methods and expression promoters. Such breadth can impose restrictions on freedom to operate, requiring licensing agreements in industrial contexts. Furthermore, there is a discrepancy between the number of patents filed and the products available at commercial scale. Much of the technology described remains at the proof-of-concept or pilot study stage, not yet reaching industrial maturity, with technical challenges such as sgRNA expression control, editing system efficiency, and genetic stability still to be overcome [50].

Overall, the growing number of patents involving CRISPR/Cas-edited *Y. lipolytica* demonstrates the dynamism and potential of this field for industrial biotechnology. However, critical analysis reveals that, although intellectual property protection is an important step to foster innovation, its effective translation into accessible products still depends on overcoming technical, regulatory, and economic barriers.

### Main CRISPR/Cas platforms in ***Y. lipolytica***

Among the *Y. lipolytica* strains used in recombinant system construction through CRISPR/Cas technologies, the W29 strain stands out as one of the most widely used genetic backgrounds. Originally isolated from sewage water in France and later characterized in 1973, W29 is a wild-type, prototrophic strain of mating-type A that has served as the parental strain for several genetically engineered derivatives. Its ability to efficiently secrete recombinant proteins and the availability of its fully sequenced genome have made it a valuable platform for biotechnological applications and genome engineering studies [3, 51, 52]. Based on the favorable phenotype of W29 ura3 mutants for protein secretion, the Po1 strain series was subsequently developed through targeted genetic modifications introducing uracil and leucine auxotrophies. These modifications facilitated the use of selectable markers and simplified genetic manipulation in metabolic engineering experiments [53]. For many years, Po1d has been one of the most widely used host strains in *Y. lipolytica* research. Another frequently used chassis strain is Po1f, which contains deletions in extracellular protease genes. The removal of these proteases reduces proteolytic degradation of heterologous proteins, thereby improving product stability, structural integrity, and overall yield during recombinant protein production. Additional strains in the Po1 series include Po1h, which is auxotrophic only for uracil, Po1t, a prototrophic derivative, and Po1g, which contains a bacteria-derived pBR322 docking platform that facilitates targeted integration of plasmid constructs. However, the presence of bacterial sequences in Po1g may affect its GRAS (Generally Recognized as Safe) status in certain regulatory contexts. Beyond the Po1 lineage, the JMY strains, derived from Po1d, are also widely used in metabolic engineering studies due to their suitability for pathway optimization and recombinant protein production [1].

To support genome engineering in *Y. lipolytica*, several vector systems and CRISPR-based platforms have been developed. One commonly used backbone is the plasmid pMCS-Cen1, which enables the insertion of guide RNA (gRNA) sequences and selectable markers such as *URA3*, facilitating targeted genome modification [54]. Other frequently used systems include pCRISPR-hph-sg [21], which carries a hygromycin resistance marker, and pCRISPRyl-AXP [55], a vector assembled using the AvrII restriction enzyme derived from *Anabaena variabilis*. Additional CRISPR systems, including piCAS9, pCASyl, peCASyl, and pCAS1yl, are based on engineered Cas9 variants designed to enhance editing efficiency in *Y. lipolytica* [24, 55]. These vectors typically incorporate codon-optimized Cas9 genes, nuclear localization signals, and selectable markers based on auxotrophic complementation or antibiotic resistance.

The choice between episomal plasmid-based systems and genome-integrated Cas9 platforms represents an important strategic decision in genome engineering in *Y. lipolytica*. Although several CRISPR/Cas9 tools have been developed for this yeast, the literature still provides limited critical discussion regarding the practical trade-offs associated with each approach [16, 56]. Episomal systems enable transient expression of Cas9 and gRNA, allowing rapid editing cycles and subsequent removal of the CRISPR machinery once selective pressure is withdrawn. This feature is particularly advantageous in studies that require the rapid construction and screening of multiple genetic variants [57]. Moreover, transient expression may reduce the likelihood of off-target effects and limit the metabolic burden imposed on the host cell. However, these systems also present notable limitations, including plasmid instability, variation in plasmid copy number, and the need for continuous selective pressure to maintain plasmid retention within the cell population. Such factors may introduce experimental variability and reduce reproducibility across independent experiments, while potentially increasing operational costs in large-scale applications. In contrast, genome-integrated Cas9 platforms provide stable nuclease expression, which can enhance editing efficiency and improve experimental consistency. These systems are therefore particularly attractive for metabolic engineering strategies that require multiple sequential edits or coordinated modifications of entire metabolic pathways [56]. Nevertheless, this approach also has limitations. Constitutive Cas9 expression may increase the risk of unintended DNA cleavage and impose an additional metabolic burden on the host cell, especially in strains that have already undergone extensive genetic modification [57]. In addition, permanent integration of the CRISPR machinery may reduce experimental flexibility in subsequent rounds of genetic engineering. Overall, the selection of episomal or genome-integrated systems should be guided by the specific objectives of the engineering strategy. Episomal plasmids are generally better suited for rapid strain construction and preliminary screening, whereas genome-integrated Cas9 platforms are often preferred for long-term metabolic engineering.

Among the CRISPR editing systems, the plasmid pMEG_YLCas9_leu2_gRNA_A, assembled via Golden Gate Assembly, represents an important platform by enabling the expression of guide RNAs under specific promoters while employing LEU2 as a selectable marker. In addition to Cas9-based systems, alternative nucleases such as Cpf1 (Cas12a) have expanded the genome engineering toolbox. Vectors including LbCpf1 and pYLXP’-AsCpf1, derived from *Lachnospiraceae bacterium* ND2006, recognize PAM sequences distinct from those targeted by Cas9, thereby allowing editing at additional genomic loci [58]. Modular CRISPR platforms have also been developed to facilitate the identification of edited clones and streamline strain construction. For instance, Golden Gate–assembled systems such as GGA-NATex-CrisprCas9-yl-RFP and GGA-HPHex-CrisprCas9-yl-RFP integrate selectable markers and reporter genes into the editing constructs. In these systems, NAT confers resistance to nourseothricin and HPH confers resistance to hygromycin B, while the inclusion of red fluorescent protein (RFP) enables visual screening of transformed strains [2, 56]. Additional plasmids are commonly employed to support gene expression and targeted genome integration in *Y. lipolytica*. Examples include pSC012, which incorporates auxotrophic markers such as URA3 or LEU2, and plasmids derived from the pINA system, such as pINA1269 and pINA1312, which maintain stable low-copy expression due to their centromeric origins of replication. Similarly, pHR_A08_Cas9 is frequently used in metabolic engineering studies, while the pCasNA-Int series (IntB11, IntC2, IntD12, and IntE8) has been designed to promote targeted integration at defined genomic loci, facilitating both genome editing and gene overexpression. Beyond individual plasmids, several modular toolkits have been developed to support the construction of complex metabolic pathways. Platforms such as YaliBricks enable hierarchical assembly of multigene pathways, whereas complementary systems like EasyCloneYALI simplify cloning and genomic integration strategies for metabolic engineering applications. In addition, hybrid vectors such as pUC19_HMG1_GND1_LEU2 combine a high-copy bacterial backbone with genes of interest and selectable markers, supporting plasmid propagation in bacteria and gene expression or complementation studies in yeast [2, 58].

Taken together, the diverse plasmid systems and modular toolkits described above have significantly expanded the genetic engineering capabilities of *Y. lipolytica*. However, the effective implementation of these platforms depends not only on the availability of molecular tools but also on the accurate design of guide RNAs and editing strategies. Bioinformatics approaches have become essential for supporting CRISPR-based genome engineering.

### Bioinformatics tools for designing crispr platforms in ***Y. lipolytica***

Bioinformatics tools have proven indispensable for the design and construction of efficient CRISPR methodologies, enabling the analysis of genomic sequences, the prediction of cleavage sites, and the evaluation of potential off-targets. These digital platforms support the selection of highly specific RNA orientations, vector modeling, and the optimization of editing strategies, reducing experimental time and increasing the efficiency and safety of genetic interventions. Thus, the integration of bioinformatics tools into the design of CRISPR platforms represents a crucial advance for the development of more precise and robust genetic engineering applications [1, 19]. In *Y. lipolytica*, different computer tools were used to construct standardized CRISPR platforms, and will be discussed below.

### Bioinformatics tools for web-integrated gRNA design

The guide RNA (gRNA) is essential for the effectiveness of the CRISPR/Cas system as it directs the Cas nuclease to the specific genomic target. The rational design of gRNAs is crucial to maximize on-target activity and reduce off-target effects. This process relies heavily on bioinformatics tools that utilize genomic data and predictive algorithms [59]. Several platforms have been developed to facilitate gRNA design, differing in analytical scope, support for specific nucleases, and interfaces. These platforms can be web-integrated, commercially available, or standalone [60, 61]. Choosing the right tool directly impacts the efficiency and specificity of genome editing. Web-based tools frequently serve as the initial point of access for gRNA design due to their inherent accessibility and user-friendly interfaces. These platforms typically integrate target identification, on-target activity scoring, and off-target prediction into a streamlined workflow. Table 1 provides a comparative overview of web-integrated platforms.

Among the diverse array of bioinformatics tools available, DeepGuide holds a unique and critical position for research in *Y. lipolytica*. It is currently the only predictive tool developed using a deep learning model trained exclusively on large-scale, empirical CRISPR screening data from *Y. lipolytica* itself [86]. This species-specific training is paramount, as prediction models developed on data from other organisms, such as human cells or *E. coli*, have demonstrated poor performance in forecasting gRNA activity in yeast due to significant differences in genomic context and DNA repair mechanisms. DeepGuide provides highly accurate predictions for on-target gRNA activity for both Cas9 and Cas12a nucleases, achieving Pearson correlation coefficients that significantly surpass those of generic, non-specialized models. The development of DeepGuide was uniquely possible due to the increasing availability of high-quality genomic sequencing and robust empirical datasets specifically for *Y. lipolytica.*

The tool is implemented as a command-line utility, requiring users to install it locally and run predictions via a Python script. While this presents a steeper learning curve compared to web-based platforms, the superior accuracy it offers is indispensable for experiments demanding high-efficiency editing, such as genome-wide screens or complex metabolic engineering projects. Although DeepGuide stands out as the only tool specifically trained on *Y. lipolytica* empirical data, it is not the only option available for researchers. Other bioinformatics tools, which utilize more generalized predictive models, offer distinct advantages in terms of accessibility, user-friendly interfaces, and integration with broader molecular biology workflows. Therefore, these tools remain highly relevant for routine editing tasks and initial target identification, as will be addressed in the following sections.

**Table 1:** Comparison of major integrated platforms for gRNA design

Tool	Platform	Key Features	Y. lipolytica Support	Cost
CHOPCHOP v3	Web & API	Supports multiple nucleases (Cas9, Cas12a, etc.); On-target and off-target scoring.	Direct. Genome is selectable from a list.	Free (Academic)
CRISPOR	Web	Aggregates multiple on-target scoring algorithms; Primer design for cloning.	Partial. Requires genome FASTA upload.	Free (Academic)
Benchling CRISPR Tool	Web	Integrated with a full molecular biology suite; Project and sequence management.	Yes. Custom genome upload.	Free for Academics
Synthego/IDT Tools	Web	Streamlined design-to-order workflow for synthetic gRNAs.	Limited. Requires sequence input; not genome-wide.	Design is free; cost is for reagents.

CHOPCHOP, for instance, is a widely utilized tool for gRNA design, offering support for various Cas nucleases, including the commonly employed SpCas9 and Cas12a (Cpf1). Its primary strengths lie in its versatility and direct support for the *Y. lipolytica* genome, which can be selected from a dropdown menu or uploaded as a custom FASTA file. The tool furnishes scores for predicted on-target efficiency and identifies potential off-target sites across the genome [10]. Similarly, CRISPOR presents a comprehensive suite of features, distinguishing itself by aggregating scores from multiple on-target prediction algorithms, including the widely cited Doench et al. [11] and Moreno-Mateos et al. [12] models. This aggregation enables researchers to compare predictions and make more informed decisions. Although CRISPOR does not offer native support for the *Y. lipolytica* genome in the same manner as CHOPCHOP, it readily accommodates custom genome uploads, thereby functioning as a powerful and flexible option [79]. Benchling provides a CRISPR tool seamlessly integrated within a broader, cloud-based molecular biology platform. Its principal advantage is the cohesive integration of gRNA design with downstream applications such as plasmid design, sequence verification, and comprehensive project management. This feature is particularly advantageous for laboratories that employ Benchling as their primary electronic lab notebook and sequence repository. The platform’s off-target analysis capabilities are robust, and it supports custom genome uploads [63]. Commercial entities such as Synthego and Integrated DNA Technologies (IDT) provide user-friendly web-based tools that are directly interfaced with their synthetic gRNA and reagent ordering systems. These platforms are meticulously designed for convenience, guiding users from a target gene or sequence to a curated list of pre-designed, purchasable gRNAs. While their on-target scoring models are proprietary and optimized for their specific chemical modifications and synthesis processes, they offer a rapid and straightforward pathway for researchers who prefer synthetic guides over plasmid-based expression. However, it is important to note that their predictive accuracy may be diminished for non-model organisms like *Y. lipolytica* without specific empirical validation [62]. Beyond these integrated design platforms, several specialized tools and algorithms play pivotal roles in various facets of the CRISPR workflow, often concentrating on particular scoring models, comprehensive off-target analysis, or advanced library design strategies.

### CRISPR optimization tools for gRNA activity prediction, off-target evaluation, and sgRNA library design

Optimization tools developed to predict gRNA efficiency, evaluate potential off-targets, and guide the design of sgRNA libraries have been applied to *Y. lipolytica* (Table 2). These tools combine statistical algorithms, machine learning, and genomic analysis to increase editing accuracy. The CRISPR/Cas9 Target online predictor (CCTop) is a widely recognized web-based tool instrumental in the rapid and efficient identification of high-quality target sites for CRISPR/Cas9 systems [64]. A core feature of CCTop involves its scoring method, which meticulously considers both the position and the number of mismatches between the single guide RNA (sgRNA) and the target DNA sequences [28].

Cas-OFFinder stands as a fast and highly versatile algorithm specifically developed to identify potential off-target sites for Cas9 RNA-guided endonucleases [65]. Cas-OFFinder has been seamlessly integrated into numerous CRISPR bioinformatics platforms, including CRISPR RNGE, which further enhances its predictive accuracy for off-target effects through the incorporation of machine learning techniques [66]. This continuous evolution and adaptation of Cas-OFFinder underscore its enduring importance in addressing the dynamic requirements of CRISPR research, particularly within the expanding domains of biomedical and agricultural applications [67]. sgRNA Designer refers to a broad category of bioinformatics tools specifically developed for the optimal design of single guide RNAs (sgRNAs) in CRISPR/Cas systems. These tools are indispensable for identifying specific motifs within target sequences and subsequently designing sgRNAs that exhibit enhanced on-target activity while concurrently minimizing undesirable off-target effects [60, 68].

Recent developments in the field of sgRNA design tools include TransCRISPR, an innovative online platform designed to identify specific motifs and facilitate the design of optimal sgRNAs for diverse CRISPR/Cas9 applications [68]. Another significant contribution is GLiDe (Guide Library Designer), a web-based platform meticulously crafted for the genome-scale design of sgRNA libraries, with a particular emphasis on CRISPR interference (CRISPRi) applications [65]. The critical importance of such tools is consistently highlighted in contemporary reviews, which emphasize their indispensable role in gRNA design, off-target prediction, and comprehensive data analysis within CRISPR/Cas9 research [66]. Furthermore, the increasing integration of artificial intelligence (AI) and machine learning (ML) methodologies is progressively enhancing sgRNA design capabilities, leading to more accurate predictions and improved off-target safety profiles [69].

The Two-Step Averaging Method (TSAM) represents a computational approach primarily employed for the regression of cleavage efficiencies of single guide RNAs (sgRNAs) within CRISPR/Cas9 systems [70]. Notably, TSAM has been recognized for its capacity to improve Spearman correlation coefficients when benchmarked against other state-of-the-art methods for CRISPR/Cas9 efficiency prediction [70]. While TSAM is fundamentally a methodological approach rather than a standalone web-based tool, its underlying principles and implementations are frequently incorporated into broader sgRNA design and efficiency prediction frameworks. Although direct citations of TSAM as a primary tool in recent (2023–2025) literature are less common, its conceptual contributions persist within the ongoing development of predictive models. For instance, the continuous refinement of interpretable efficiency prediction models for CRISPR/Cas9 gene editing often builds upon the insights gained from methods like TSAM [71]. The integration of such averaging or ensemble approaches remains relevant in efforts to improve overall CRISPR specificity and on-target activity, particularly as machine learning and deep learning models become more prevalent in the field [66, 69].

Seq-DeepCpf1 represents a notable advancement in the computational prediction of on-target editing efficiencies for CRISPR/Cas12a (Cpf1) systems. This tool is distinguished as one of the pioneering efforts to employ deep learning methodologies for this specific purpose, leveraging high-throughput CRISPR experimental data [72]. Its recent applications underscore its integration into novel gRNA generation frameworks, such as the CRISPR-Variational Autoencoder (CRISPR-VAE), which aims to generate gRNA sequences with inherent efficiency awareness [73]. Furthermore, Seq-DeepCpf1 has been extensively utilized in studies focused on profiling the activities and specificities of various Cas12a variants in human cells, thereby demonstrating its sustained relevance in the comprehensive characterization and optimization of Cas12a systems [26].

DeepCRISPR is a comprehensive computational platform that unifies the prediction of sgRNA on-target activity and off-target sites within a singular deep learning framework [74]. This integrated approach offers a significant advantage over more conventional bioinformatic methods [25]. DeepCRISPR has garnered recognition as one of the most accurate tools available for gRNA design and off-target prediction, establishing it as an invaluable resource for CRISPR/Cas9 research [66, 75]. Its utility spans various applications where precise gRNA design is paramount, and it continues to serve as a critical benchmark in the ongoing development of new deep learning models tailored for CRISPR/Cas systems [75]. The protocol developed by Ramesh and Wheeldon [76] represents a significant contribution to CRISPR-based genome engineering, with a specific focus on guide RNA (gRNA) design for genome-wide CRISPR screens in *Y. lipolytica*. This protocol, detailed in Methods in Molecular Biology, furnishes researchers with comprehensive methodologies for the effective design of gRNAs, a critical prerequisite for the successful execution of high-throughput functional genomic screens in this non-conventional yeast [76].

**Table 2:** Tools and resources for optimizing the CRISPR system

Tool/Resource	Function	Key reference
CCTop, Cas-OFFinder	Exhaustive search for potential off-target sites in a reference genome.	[64, 65]
sgRNA Designer	Cas9 guide activity scoring based on Doench 2014/2016 models.	[11, 77]
TSAM	Cas9 cleavage-efficiency regression.	[70]
Seq-DeepCpf1, DeepCRISPR	Neural-network-based sgRNA activity predictors for Cas12a and Cas9.	[74, 78]
Ramesh and Wheeldon (protocol)	Strategy for genome-wide sgRNA library design in *Y. lipolytica* (Cas9 and Cas12a).	[76]
ALLEGRO (ILP algorithm)	Optimization of compact sgRNA libraries across multiple species; validated in *Y. lipolytica*.	[79]

The bioinformatics landscape for CRISPR design in *Y. lipolytica* reveals a significant divergence between computational accessibility and predictive precision. While the platforms summarized in Table 1 (e.g., CHOPCHOP, Benchling) have facilitated the adoption of CRISPR technology through intuitive graphical user interfaces (GUIs), their reliance on generalized scoring models often derived from mammalian or prokaryotic datasets presents a critical limitation. As evidenced by the comparative metrics in Table 3, these general-purpose tools typically achieve only “Moderate” predictive accuracy when applied to *Y. lipolytica*. This discrepancy suggests that universal algorithms may fail to account for the species-specific chromatin architecture, nucleosome positioning, and DNA repair biases inherent to non-conventional yeasts. Consequently, the application of these tools in complex metabolic engineering projects requires careful validation to avoid suboptimal editing efficiencies. In contrast, the emergence of species-specific tools like DeepGuide represents a significant advancement in achieving high-fidelity activity prediction. By leveraging high-quality empirical data from genome-wide screens (as detailed in Table 3), DeepGuide effectively addresses the biological nuances of *Y. lipolytica*, offering a “High” precision benchmark that is currently unmatched by non-specialized models. However, a critical analysis of the current state of the art reveals a technical implementation barrier. The requirement for local installation and command-line execution (Table 3) may limit the immediate adoption of these high-accuracy models by researchers without specialized computational training. Furthermore, the development of such specialized tools is currently restricted by the availability of large-scale functional genomic data, which remains a significant challenge for other non-conventional oleaginous yeasts.

Looking forward, the field must transition toward integrated, multi-tiered design strategies. The proposed workflow involves utilizing the organizational strengths of web-based platforms for routine gene targeting and project management, while reserving specialized, species-optimized algorithms for high-throughput applications where maximum on-target efficiency is paramount. The future of CRISPR bioinformatics in *Y. lipolytica* lies in the integration of high-accuracy predictive models into more accessible, cloud-based frameworks and the expansion of empirical datasets to encompass a broader range of non-conventional species. This systematic evolution is essential to transition CRISPR-based modifications from an empirical process into a predictable and robust genetic engineering discipline.

### Workflow recommendations

One of the most significant challenges in adapting CRISPR technology to non-model organisms such as *Y. lipolytica* is the transferability of existing gRNA prediction models. Generic predictors, which are frequently trained on datasets derived from mammalian or *E. coli* systems, often exhibit suboptimal performance in yeasts. Baisya et al. [62] empirically demonstrated a poor correlation between sgRNA activities in *Y. lipolytica* and scores generated by algorithms trained on human, mouse, or *E. coli* data. This observed discrepancy is attributable to fundamental differences in genomic context, including nucleosome positioning, GC content, and DNA repair pathway biases, between yeast and mammalian genomes [62]. Addressing this critical challenge, DeepGuide represents a substantial advancement. This deep learning model was meticulously trained on empirical genome-wide CRISPR screens conducted specifically in *Y. lipolytica*. It achieved Pearson correlations of approximately 0.5 for Cas9 and 0.66 for Cas12a between predicted and observed activity, significantly outperforming other generic models. This underscores the paramount importance of species-specific training data for accurate gRNA activity prediction in non-model organisms [62]. The selection of an optimal gRNA design tool is a multifaceted decision, necessitating a careful balance among predictive accuracy, user-friendliness, and financial implications. Table 3 describes the accuracy of the tools within the specific context of *Y. lipolytica.*

**Table 3:** Comparative analysis of CRISPR tools based on accuracy, ease of use, and cost

Tool	Accuracy	Ease of Use	Cost
CHOPCHOP v3	Moderate to High. Relies on general scoring models, which may not be perfectly optimized for *Y. lipolytica*. However, its off-target analysis is robust.	High. Intuitive web interface with direct *Y. lipolytica* genome support, simplifying the design process.	Free. Academic, open-access tool.
CRISPOR	Moderate to High. Similar to CHOPCHOP, it utilizes multiple scoring algorithms that enhance prediction, but are still general in nature.	High. Clear and functional web interface. The requirement for FASTA genome upload is an additional step, but the process is well-documented.	Free. Academic, open-access tool.
Benchling CRISPR Tool	Moderate to High. Offers good off-target analysis and sequence integration. On-target activity prediction is based on general models.	Very High. Integrated into a comprehensive molecular biology platform, facilitating end-to-end project management. Polished and professional interface.	Free for academics. Paid plans for industrial/commercial use with additional features.
Synthego/IDT Tools	Moderate. Commercial tools focused on gRNA synthesis. Their scoring models are proprietary and optimized for their products, but may lack precision in non-model organisms like *Y. lipolytica* without empirical validation.	Very High. Designed to be extremely user-friendly, guiding the user from design to ordering synthetic gRNAs.	Design is free. Cost is for reagents.
DeepGuide	Very High (for *Y. lipolytica*). The most accurate tool for predicting gRNA activity in *Y. lipolytica*, as its model was specifically trained on large-scale empirical data from this yeast.	Low to Moderate. Requires software installation (Python) and command-line familiarity. Lacks a graphical web interface, posing a barrier for non-computational users.	Free. Code and model are openly available.

For genome editing in *Y. lipolytica*, an effective workflow may combine the accessibility of web-based platforms with the precision of specialized algorithms. As an initial step, it is advisable to generate a list of gRNA candidates using platforms such as CHOPCHOP [80, 81] or CRISPOR [10], which provide robust off-target analysis capabilities. These candidates can then be reassessed and prioritized with DeepGuide, a deep-learning algorithm designed to predict gRNA activity specifically in *Y. lipolytica* [62, 82]. For academic projects where usability is prioritized, Benchling offers an integrated and user-friendly environment, combining gRNA design with sequence management and cloning functions, making it a practical choice for laboratories already relying on its resources [63]. When convenience and rapid access to synthetic gRNAs with reasonable activity scores are preferred, commercial platforms such as Synthego (Synthego, n.d.) and IDT (IDT, n.d.) provide straightforward solutions, though the associated reagent costs should be considered. Across all scenarios, rigorous verification of potential off-target effects remains a critical step. Tools such as CHOPCHOP, CRISPOR, and Benchling include comprehensive off-target analysis modules and are strongly recommended for this purpose.

The selection of the most appropriate bioinformatics tool for gRNA design in *Y. lipolytica* is inherently contingent upon specific project objectives, the availability of resources, and the desired level of precision. Effective genome editing in *Y. lipolytica* utilizing CRISPR technology necessitates a strategic selection of bioinformatics tools, meticulously tailored to specific project requirements and precision demands. For research endeavors demanding the utmost precision, particularly within *Y. lipolytica*, an optimal workflow integrates user-friendly web-based tools with highly specialized algorithms. This methodological approach typically commences with the generation of an initial roster of single guide RNA (sgRNA) candidates using established platforms such as CHOPCHOP v3 [19, 81] or CRISPOR [10]. These tools are invaluable for their robust capabilities in predicting and analyzing potential off-target effects. Subsequently, these candidate sgRNAs can undergo a rigorous re-scoring and prioritization process using DeepGuide, a sophisticated deep learning algorithm specifically trained on *Y. lipolytica* data to accurately predict guide activity [82]. For academic projects where ease of use and integrated workflows are paramount, Benchling offers a highly intuitive and comprehensive experience. This platform seamlessly combines gRNA design with broader molecular biology functionalities, encompassing sequence management and cloning, thereby making it an excellent choice for laboratories already integrated into its ecosystem [63]. When the primary objective is convenience and the rapid procurement of synthetic sgRNAs with reasonable activity scores, direct options are readily available through commercial providers. Tools offered by Synthego (Synthego, n.d.) and IDT (IDT, n.d.) facilitate expedited design and ordering, although the associated reagent costs warrant careful consideration. Irrespective of the chosen strategy, rigorous off-target verification remains an indispensable step in all CRISPR genome editing scenarios. Tools such as CHOPCHOP v3, CRISPOR, and Benchling are equipped with comprehensive off-target prediction capabilities and are highly recommended for this critical analysis.

### Limitations and challenges in crispr-based gene editing in ***Y. lipolytica***

Despite the transformative potential inherent in CRISPR/Cas systems, their application within *Y. lipolytica* faces challenges. These limitations frequently arise from the intrinsic biological complexities of the organism itself, coupled with the computational hurdles encountered in accurately predicting gRNA efficacy and specificity. Fundamentally, the accuracy of gRNA design tools is predicated upon the quality and completeness of the reference genome and its associated annotation. For *Y. lipolytica*, while several genome assemblies are available, variations in annotation can lead to discrepancies in target identification and subsequent off-target prediction. Incomplete or erroneous gene models can, in turn, result in gRNAs inadvertently targeting non-coding regions or eliciting unintended effects [7]. Minimizing off-target cleavage continues to represent a significant challenge. Even with the deployment of highly sophisticated algorithms, predicting all potential off-target sites with absolute certainty remains arduous, particularly in organisms characterized by repetitive sequences or elevated genomic plasticity. Such off-target effects can precipitate unintended mutations, thereby impacting cellular viability, metabolic pathways, or the desired phenotypic outcomes [83]. As conspicuously highlighted by the development of DeepGuide, generic gRNA activity prediction models frequently demonstrate suboptimal performance in *Y. lipolytica* due to species-specific genomic features and distinct DNA repair mechanisms. While DeepGuide specifically addresses this deficiency for *Y. lipolytica*, the continuous evolution of CRISPR/Cas variants and the emergent need for novel nucleases (e.g., base editors, prime editors) necessitate the ongoing development of new, empirically-trained prediction models [62].

Efficient delivery of CRISPR components (comprising the Cas protein, gRNA, and donor DNA) into *Y. lipolytica* cells can often constitute a significant bottleneck. Although various methodologies exist (e.g., electroporation, lithium acetate transformation), their efficiency considerably depending on the specific strain, target gene, and prevailing experimental conditions, thereby influencing the overall success rate of gene editing [2, 19]. Furthermore, the design and implementation of multiplexed gene editing strategies aiming to edit multiple genes concurrently in *Y. lipolytica* introduce additional layers of complexity. This encompasses the optimization of multiple gRNAs, ensuring balanced expression of all constituent components, and adeptly managing the increased potential for off-target effects and unforeseen interactions between distinct editing events [84].

To maximize both the success rate and specificity of CRISPR-based gene editing in *Y. lipolytica*, researchers are advised to adopt several practices. Foremost among these is the prioritization of tools such as DeepGuide, which have been specifically trained and rigorously validated on *Y. lipolytica* datasets for gRNA activity prediction. In instances where such specialized tools are unavailable for novel Cas systems, the development of custom models based on empirical data derived from *Y. lipolytica* should be considered [62]. It is imperative to consistently perform thorough off-target analysis utilizing multiple bioinformatics tools (e.g., CHOPCHOP, CRISPOR, Benchling) to proactively identify and minimize potential off-target sites. For applications deemed critical, experimental validation of off-target effects, for example, through whole-genome sequencing, is highly recommended [83]. Ensuring the use of the most current and comprehensively annotated *Y. lipolytica* genome assembly for gRNA design is also crucial. If working with a novel strain, sequencing and annotating its genome can significantly enhance the accuracy of predictions [7]. Notwithstanding advancements in predictive algorithms, empirical validation of gRNA efficiency and specificity in *Y. lipolytica* remains paramount. This typically involves screening multiple gRNA candidates for each target gene and meticulously assessing their on-target editing rates and potential off-target effects through methods such as Sanger sequencing or next-generation sequencing [62]. Furthermore, experimenting with diverse CRISPR component delivery methods and transformation protocols is essential to identify the most efficient approach tailored to specific *Y. lipolytica* strains and experimental setups. Factors such as cellular growth phase, electroporation parameters, and donor DNA concentration can profoundly impact transformation efficiency [2, 19]. For multiplexed editing or library screening, the employment of advanced algorithms like ALLEGRO or adherence to established protocols [76] for optimizing gRNA libraries is recommended. This strategic approach helps ensure the generation of compact and efficient libraries while concurrently minimizing unintended interactions and off-target effects [76, 79].

### Future trends and perspectives

Several limitations discussed in the previous section highlight important opportunities for advancing CRISPR-based genome engineering in *Y. lipolytica*. In particular, improving gRNA activity prediction, reducing off-target effects, and increasing editing efficiency will require both methodological innovations and the generation of new experimental datasets specifically tailored to this yeast.

One promising direction involves the application of artificial intelligence and machine learning (AI/ML) approaches to improve gRNA design. However, the development of accurate predictive models for *Y. lipolytica* will depend on the availability of large, high-quality experimental datasets. These may include the availability of reference genomes for different *Y. lipolytica* strains, as well as genome-wide CRISPR screening datasets measuring gRNA activity, systematic analyses of off-target cleavage events, and datasets describing DNA repair outcomes following CRISPR-induced double-strand breaks. In addition, incorporating genomic features such as chromatin accessibility, nucleosome positioning, and local sequence context could significantly improve the predictive performance of future models. The success of DeepGuide demonstrates the potential of species-specific approaches; however, further model refinement will likely require expanded datasets generated under diverse growth conditions and across multiple *Y. lipolytica* strains. Advances in bioinformatics are also expected to play a key role in overcoming current limitations in CRISPR system design. Future computational tools are likely to incorporate multi-omics datasets directly into the gRNA design pipeline. In the case of *Yarrowia lipolytica*, this integrative approach may combine genomic information with transcriptomic, proteomic, and metabolomic data to provide deeper insights into gene regulatory networks and metabolic pathway dynamics. Such strategies may enable more informed target selection for metabolic engineering, particularly in the context of lipid metabolism and industrial bioproduction, thereby supporting the development of more rational and efficient CRISPR-based engineering strategies [41, 85, 86].

Emerging genome editing technologies also offer opportunities to address some of the limitations associated with conventional CRISPR nucleases. In particular, base editing and prime editing systems may enable precise nucleotide-level modifications without inducing double-strand breaks. However, the implementation of these tools in *Y. lipolytica* will require systematic evaluation of editing efficiency, PAM compatibility, and DNA repair outcomes in this species. The generation of comprehensive editing efficiency maps across different genomic loci may facilitate the rational selection of editing strategies for metabolic engineering applications.

Delivery of CRISPR components remains a crucial technical challenge in many genome engineering applications. Transformation efficiency can vary considerably depending on the strain, the physiological state of the cells, and experimental conditions, often limiting editing efficiency and experimental reproducibility. In this context, future research is likely to focus on the development of more efficient and less cytotoxic delivery strategies. Promising approaches include nanoparticle-based delivery systems, such as lipid or polymeric nanoparticles capable of transporting CRISPR ribonucleoproteins (RNPs) directly into cells, thereby reducing dependence on plasmid-based vectors and minimizing off-target effects associated with prolonged nuclease expression. In addition, the optimization of electroporation protocols specifically for *Y. lipolytica*, for example by adjusting parameters such as pulse intensity, medium conductivity, and cellular growth phase, may significantly improve transformation efficiency. Other potential strategies include the development of protoplast-based transformation systems, the use of integrative vectors with stronger or regulatable promoters, and the adaptation of delivery methods previously developed for other yeasts or filamentous fungi. Improvements in these delivery systems will be particularly important for multiplex genome editing applications, large-scale genomic screens, and the development of industrial *Y. lipolytica* strains with optimized metabolic characteristics [2, 18, 19].

The integration of computational CRISPR design tools with automated experimental platforms is also expected to significantly accelerate metabolic engineering workflows in *Y. lipolytica*, enabling automated pipelines for gRNA design, robotic assembly of CRISPR constructs, and high-throughput phenotypic screening of edited strains within the design–build–test–learn (DBTL) cycle. Continued advances in specialized bioinformatics tools, more precise genome editing systems, and integrated experimental platforms will be essential to overcome current limitations and further establish *Y. lipolytica* as a robust chassis for applications in industrial biotechnology and metabolic engineering [84].

## Supplementary Information

Below is the link to the electronic supplementary material.

Supplementary Material 1

## Data Availability

No datasets were generated or analysed during the current study.
