# Supplementary material for: 10 years of CRISPR/CAS genomic engineering in Yarrowia lipolytica
Source: Bioprocess Biosyst Eng. 2026 May 23;49(7):1767–83. doi: 10.1007/s00449-026-03347-1 (PMC13379446; doi:10.1007/s00449-026-03347-1)
Supplement: Supplementary file 1 — Supplementary Material 1 [file 449_2026_3347_MOESM1_ESM.docx]

**10 YEARS OF CRISPR/CAS GENOMIC ENGINEERING IN *Yarrowia lipolytica***

***Bioprocess and Biosystems Engineering***

Rodrigo Gonçalves Dias^1,2^, Fernanda Pinheiro Moreira Freitas^1,2^, Samuel Lessa Barbosa^1,2^, João Victor Marques Gonçalves Assis^1,2^, Thaynara Lorenzoni Entringer^1,2^, Juliana Silva Carneiro Fonseca^1,2^, Miguel Edmundo Romanizio^1,2^, Bruno Brayan Zanotti Pimentel^1,2^, Maria Emilene Martino Campos-Galvão^1,2^, Nívea Moreira Vieira^1,2^, Luciano Gomes Fietto^1,3^, Agustin Zsögön^4^, Wendel Batista da Silveira^1,2^.

*^1^* Department of Microbiology, Universidade Federal de Viçosa, Viçosa, MG, 36570-900, Brazil

*^2^* Institute of Biotechnology Applied to Agriculture (BIOAGRO) - Universidade Federal de Viçosa, Viçosa, MG, 36570-900, Brazil

*^3^* Department of Biochemistry and Molecular Biology, Universidade Federal de Viçosa, Viçosa, MG, 36570-900, Brazil

*^4^* National Institute of Science and Technology on Plant Physiology Under Stress Conditions, Department of Plant Biology, Universidade Federal de Viçosa, Viçosa, MG, 36570-900, Brazil

Corresponding author: Wendel Batista da Silveira

E-mail address: [wendel.silveira@ufv.br](mailto:wendel.silveira@ufv.br)

Supplementary material 1: CRISPR/Cas Genome Engineering in *Yarrowia lipolytica*

| ***Y. lipolytica* strain** | **CRISPR System** | **Plasmid/Casset** | **Delivery of components** | **Repair mechanism** | **Genetic modification** | **Objective/product** | **Reference** |
| --- | --- | --- | --- | --- | --- | --- | --- |
| PO1f | CRISPR-Cas9 | pCRISPRyl | Plasmid transformation (episomal expression system) | HR | Knockout of PEX10, KU70, MFE1. Targeted integration of heterologous cassette (HPH resistance marker) | Development of a high-efficiency, markerless CRISPR–Cas9 genome editing tool for *Y. lipolytica* | <https://doi.org/10.1021/acssynbio.5b00162> |
| ATCC201249 ATCC MYA-2613  CIBTS1604  CIBTS1605  CIBTS1961 | CRISPR-Cas9 | pCAS1yl/ pCAS2yl | Transformation using SC-leu medium | HR and NHEJ | Knockout of TRP1, PEX10 and GUT2 | Demonstrate a single-plasmid CRISPR-Cas9 system (pCASyl) for efficient gene disruption in *Y. lipolytica* | [.](https://doi.org/10.3389/ffunb.2024.1327777)<https://doi.org/10.1007/s10295-016-1789-8> |
| PO1f | CRISPR-Cas9 | UAS1B8-TEF-cas9-CYC1 + TEF-HH-sgRNA-HDV  UAS1B8-TEF-cas9-CYC1 + TEF-HH-(XDH)sgRNA-HDV  UAS1B8-TEF-cas9-CYC1 + SCRp’-tRNAp-(PEX10)sgRNA  UAS1B8-TEF-cas9-CYC1 + SCRp’-tRNAp-(XKS)sgRNA | Lithium acetate transformation | HR and NHEJ | Gene deletion of overexpression of XDH, XYR1, XYR2 and XKS genes | Study xylose pathway of *Y. lipolytica* | <https://doi.org/10.1186/s13068-016-0562-6> |
| ATCC 201249 ATCC MYA-2613  CIBTS1776 | CRISPR-Cas9 | pCen1-Cas9  pCAS1yl-trp/ura3 | Zymogen Frozen-EZ yeast transformation kit II (Zymo Research Corporation) (Blazeck et al. | - | heterologous multifunctional β-carotene synthase containing phytoene synthase, phytoene dehydrogenase, and lycopene b-cyclase activities encoded by carS from Schizochytrium sp. | Production of β-carotene | <https://doi.org/10.1007/s10529-017-2318-1> |
| *Y. lipolytica* Po1g ΔLeu | CRISPR-Cas9 | pYaliA1-hCas9 along with CAN1 sgRNA PCR product | Lithium acetate transformation | non-homologous end joining (NHEJ) or homology-directed repair (HDR) | 12 kb five-gene violacein biosynthetic pathway | Create a genetic toolkit for Y.l. | <https://doi.org/10.1016/j.meteno.2017.09.001> |
| PO1f | CRISPR-Cas9 and dCas9-Mxi1 and dCas9 | pCRISPRi_Mxi1_yl_NHEJ, pCRISPRyl | - | HR | PEX10 | Make a system for repress the NHEJ using dCas9 while you can perform a normal CRISPR for increase the efficiency of HR | <https://doi.org/10.1002/bit.26404> |
| GB20 | CRISPR-Cas9 | pCfB4906, sgRNA/gRNA vectors, marker-free integration cassettes, prototrophic marker integrative vectors | Lithium acetate transformation | Homologous recombination using non-replicating repair templates | Gene deletions of URA3, LEU2, ALK1, and GUT2, marker-free integration of expression cassettes at IntB, and precise point mutations. | Marker-free genome editing in *Y. lipolytica* with >80 % efficiency for integrations and knockouts; enable rapid strain construction for metabolic engineering | ]<https://doi.org/10.1002/biot.201700543> |
| Typical *Y. lipolytica* laboratory strain (not specified) | CRISPR-dCas9 activation (CRISPRa) using synthetic tripartite activator VPR fused to dCas9 | dCas9-VPR fusion vector ( pCRISPRa_VPR_yl) with an AvrII site for sgRNA insertion | Likely transformed via standard yeast transformation (LiAc/PEG)—not explicitly detailed in abstract | Transcriptional activation (no DNA cleavage or repair) | Activation (upregulation) of native β-glucosidase genes and activation of a synthetic promoter driving hrGFP | Enable growth on cellobiose by upregulating cryptic sugar-metabolism genes; synthetic-biology toolbox expansion | <https://doi.org/10.1007/978-1-4939-7795-6_18> |
| PO1f | CRISPR-Cas9 | pIW771, pCRISPRyl | Lithium acetate transformation/PEG/heat-shock with plasmid sgRNA library | NHEJ; HR | KU70 knock out; CAN1 deletion (canavanine resistance), knockouts that enhance lipid accumulation, and knockouts that eliminate pseudohyphal morphology | identify essential genes (1,450 ORFs), characterize determinants of sgRNA efficiency, and discover phenotypes such as high lipid accumulation and loss of pseudohyphal growth | <https://doi.org/10.1002/biot.201700584> |
| Y6047 e PO1d | CRISPR-Cas9 | pCg58-FAD2 | Frozen EZ Yeast Transformation II kit (Zymo Research) | NHEJ e HDR | RedStar2 gene to quantify gene disruption efficiency; endogenous FAD2 gene. | optimize the efficiency of the CRISPR-Cas9 system in *Yarrowia lipolytica* by evaluating factors such as Cas9 expression levels and sgRNA maturation; promote in vivo directed mutagenesis via homology-directed repair and confirm the lack of off-target effects | <https://doi.org/10.1016/j.jmb.2018.08.024> |
| PO1f | CRISPR-Cas9 | pCas9-LEU or URA plasmids; pCRISPRyl | Lithium acetate transformation | HR, NHEJ and HMEJ | Gene excision (PEX10, FAA1, POX3, MFE, LEU2, URA3) and targeted integration (hph into PEX10 locus) | Efficient markerless deletion of genes to study peroxisome function and lipid accumulation; targeted integration of heterologous genes | <https://doi.org/10.1002/biot.201700590> |
| DSM-3286 and DSM-21 175 | CRISPR-Cas9 | pMEG_YLCas9_leu2_gRNA_A  (GOLDENMOCS) | Lithium acetate transformation | NHEJ | Knockout of LEU2 | They used a new plasmid constructed by their GoldenMOCS method (a variation of GoldenGate) to generate leucine auxotrophic strains (apparently not laboratory strains). Proof of concept. | <https://doi.org/10.1093/femsle/fnz022> |
| pO1g | CRISPR-Cas9 | PYaliA1 | Lithium acetate transformation | NHEJ | Knockout of CAN1 | Proof of concept to demonstrate the use of the expression system | <https://doi.org/10.1007/978-1-4939-9142-6_11> |
| PO1f and PO1f/ku70Δ | CRISPR-Cas9 | pCRISPRyl | Lithium acetate transformation | NHEJ/HR | All genes were tested | Generation of a yeast library with almost all genes modified by different gRNAs in order to generate a library, test different gRNAs, compare the methodology with E. coli, and discover essential genes. | <https://doi.org/10.1016/j.ymben.2019.06.007> |
| PO1f | CRISPR-Cas9 | pCRISPRyl | Lithium acetate transformation | NHEJ | - | Testing of different nucleotides  in intergenic regions between sgRNA and tRNA | <https://doi.org/10.1016/j.ymben.2020.07.008> |
| Po1g1f e Po1g | CRISPR/Cas12/Cpf1 | pYLXP'-AsCpf1 | Lithium acetate transformation | NHEJ | knockout of CAN1, URA3 e MET25/MET2/MET6 | Standardization of a protocol for transformation using the CRISPR/Cas12 system for both single and multiple genes simultaneously. This enzyme recognizes other cleavage sites and generates cohesive ends at the cut. | <https://doi.org/10.1016/j.mec.2019.e00112> |
| w29 | CRISPR-Cas9 | pCRISPR-hph-sg YlACL2 | Electroporation | NHEJ | knockout of YlACL2 | Comparison between convencional method and CRISPR-Cas9 for gene deletion | <https://doi.org/10.1134/S0003683820090112> |
| IMUFRJ 50682 | CRISPR-Cas9 | GGA_NATex_CRISPR-Cas9Cas9-yl_RFP and GGA_HPHex_CRISPR-Cas9Cas9-yl_RFP (GOLDEN GATE) | Plasmid transformation by lithium-acetate | NHEJ | knockout of URA3 | Generation of auxotrophic strains | <https://doi.org/10.1016/j.enzmictec.2020.109621> |
| Po1g and Po1g/ku70Δ | CRISPR/d/nCas9 | pCRISPRyl | Plasmid transformation by lithium-acetate | NHEJ/HDR | Knockout of TRP1, PEX10 and HIS3 | They created a TARGET-AID system in yeast using a Cas9-coupled deaminase for C-to-T substitution, tried it on three genes and on these genes at the same time, also varying the yeast strain. | <https://doi.org/10.1002/biot.201900238> |
| CLIB122 | CRISPR/Cpf1 (Cas12) | LbCpf1 | Plasmid transformation by lithium-acetate | NHEJ | MGA1, CAN1 e URA3 | They developed a CRISPR system that can delete, repress, or activate genes. This system can delete multiple genes with good efficiency or delete and activate/repress using different sizes of gRNA and presence of transcription factor in Cas9. | <https://doi.org/10.1021/acssynbio.9b00498> |
| W29, Po1d  , Po1dΔku70 and wildtype strains | CRISPR-Cas9 | CRISPRyl | Plasmid transformation by lithium-acetate | NHEJ/HR | EYK1 , EYD1, URA3, MFE2 , GSY1 and LIP2 | Testing the manufacture of different vectors using golden gate to produce auxotrophic wild strains and how each auxotrophy influences CRISPR efficiency | <https://doi.org/10.1007/s10529-020-02805-4> |
| ACCDGA | CRISPR-Cas9 | CRISPRyl | Plasmid transformation by lithium-acetate | NHEJ | Knockout of PEX10 | In the context of bgal expression and overexpression of galactose genes to consume lactose from acid whey, researchers used CRISPR only to delete PEX10. | <https://doi.org/10.1016/j.ymben.2019.09.010> |
| PO1f | CRISPR-Cas9 | CRISPRyl | - | HR | Insertion of XR , ss XDH e yl XK | They used CRISPR to insert the three xylose metabolism genes into the genome with the aim of using hydrolysate to generate limonene. | <https://doi.org/10.1093/femsyr/foaa046> |
| - | - | - | - | - | - | Review paper | <https://doi.org/10.1007/s10295-020-02290-8> |
| Pof1 | CRISPR-Cas9 | CRISPRyl | Frozen-EZ Yeast Transformation II Kit™ (Zymo Research Corporation, Irvine, CA) | HR | Knockin carRP, carB, GGS1, AMPD , HMG, ERG10, ERG13, ERG12, ERG8, ERG19 and IDI | Enginieer for production of -caroten | <https://doi.org/10.1007/s10529-020-02844-x> |
| ATCC 201249 | CRISPR-Cas9 | pMCS-Cen1 | Kit Zymogen Frozen EZ Yeast Transformation II (Zymo Research Corporation) | NHEJ | knockout of PXA1, PEX10, MFE1, POT1, RPD3 , SNF1, LRO1, PAH1, DGK1, MGA2 | They used CRISPR only to delete beta oxidation genes in the aim of increasing lupenol production. | <https://doi.org/10.1186/s13068-020-01773-1> |
| PO1g | CRISPR-Cas9 | pCRISPRyl | Lithium acetate transformation | – | Delete genes related to neutral fatty acid synthesis | Increase hydroxy fatty acid synthesis | <https://doi.org/10.3389/fbioe.2021.624838>[38] |
| W29_ST6512 | CRISPR-Cas9 | pCfB4906 | DNA was transformed into the parent strain using lithium acetate transformation | HR | 11 genes encompassing Moco biosynthesis, molybdate transport and nitrate reduction functions were introduced | Increase the molybdenum cofactor | <https://doi.org/10.1093/femsyr/foab050> |
| PO1d | CRISPR-Cas9 | JME 4390 | Lithium acetate transformation | NHEJ | Yl4HPPD disruption | Analyze the effects of the knockout on the polymelanin production | <https://doi.org/10.3390/microorganisms9040838> |
| PO1g | CRISPR-Cas9 | pCAS1yl | Linear plasmid lithium acetate transformation | HR | Disruption of the PLA2-3 gene | Explore the effects of PLA2 on lipid accumulation | <https://doi.org/10.1111/jam.14779> |
| PO1f | CRISPR-Cas9 | pCAS1yl-trp | Lithium acetate transformation | Homology-independent integration | - | Optimization of a homology-independent targeted genome integration tool mediated by CRISPR-Cas9 | [https://doi.org/10.1128/AEM.02666‑2](https://doi.org/10.1128/AEM.02666%E2%80%9120) |
| PO1f | CRISPR-Cas9 | pHR | Lithium acetate transformation | HR | Integration and expression of novel diamine oxidase genes | Production of DAO to degrade histamin. | <https://doi.org/10.1016/j.jbiotec.2021.08.015> |
| ATCC 201249 | CRISPR-Cas9 | pMCS-Cen1 | Zymogen Frozen EZ Yeast Transformation Kit II | NHEJ | Acyl-CoA reductase (FAR) gene and gene A1 (YlΔA1-FAR) | Increased production of fatty alcohols | <https://doi.org/10.3389/fmicb.2022.898884> |
| ST9149 (derived from W29) | CRISPR-Cas9 | EasyCloneYALI | Lithium-acetate transformation | NHEJ | Genes envolvidos na biossíntese de ABA (BcABA1, BcABA2, BcABA3, BcABA4 e BcCPR1) | Production of abscisic acid | <https://doi.org/10.1093/femsyr/foac015> |
| PO1f | CRISPR-Cas9 | CRISPR-Cas9-trp-F, CRISPR-Cas9-trp-R | Lithium-acetate transformation | NHEJ | TRP1 interruption | Removal of lycopene substrate inhibition enables high carotenoid productivity | <https://doi.org/10.1038/s41467-022-28277-w> |
| DSM 3286 | CRISPR-Cas9 | pMEG_BB3_YL68N_Ac | Lithium-acetate transformation | NHEJ | YALI2C00079g (STL8) | Glycerol transport activity | <https://doi.org/10.1002/yea.3702> |
| Pof1 | CRISPR-Cas9 | pCAS1yl | Lithium-acetate transformation | NHEJ | The CRISPR–Cas9 system was used to delete DGA1 and DGA2 to obtain Q24–Q26 strains. | Production of the β-farnesene using lipid as the substrate | [https://doi.org/10.1186/s13068‑022‑02201‑2](https://doi.org/10.1186/s13068%E2%80%91022%E2%80%9102201%E2%80%912) |
| YLBI3118 | CRISPR-Cas9 | pCAS1yl | Kit Frozen-EZ Yeast Transformation II | NHEJ | *Y. lipolytica* was modified by introducing high membrane affinity variants of the carotenoid cleavage dioxygenase enzyme, PhCCD1, to increase the production of the aroma compound, β-ionone | Production of the β-ionone aroma compound from organic waste hydrolysates using an engineered *Yarrowia lipolytica* strain | [https://doi.org/10.1007/s00253‑023‑12731‑w](https://doi.org/10.1007/s00253%E2%80%91023%E2%80%9112731%E2%80%91w) |
| PO1f | CRISPR-Cas9 | YaliBricks | Kit Frozen-EZ Yeast Transformation II | NHEJ | Insertion of Pc4CL1 and VvSTS | Remodelling metabolism for high-level resveratrol production in *Yarrowia lipolytica* | <https://doi.org/10.1016/j.biortech.2022.128178> |
| PO1f | CRISPR-Cas9 | pCasyl;  piCas9 | - | HR | Genes related to lipid metabolism and peroxisomes (gut2, mfe1, pox3, pex10) were deleted. Insertion of genes for carotenoid pathway enzymes (carRA, carB, crtZ) | Efficient strains for the production of β-carotene and zeaxanthin | https://doi.org/10.1007/s00253-023-12731-w |
| PO1f | CRISPR-Cas9 | PIW(URA) | Lithium-acetate transformation | Base editor | Deletion of genes Deletion of MLS, CIT2, TRP2, PDT, ARO10 genes | Naringenin production | [https://doi.org/10.1021/acssynbio.3c00435](https://doi.org/10.3389/ffunb.2024.1327777) |
| PO1f | CRISPR-Cas9 | pCasNA-IntC2; pCasNA-IntE8; pCasNA-IntD12; pCasNA-IntB11. | Lithium-acetate transformation | HR; NHEJ suppressed | Deletion of ku70  superexpression of aro8; aro9; aat2. Superexpression of HPD1; ARO4; ARO7  Truncation within the HMG2 gene | Production of homogentisic acid (HGA) | [https://doi.org/10.1038/s42003‑023‑05202‑5](https://doi.org/10.1038/s42003%E2%80%91023%E2%80%9105202%E2%80%915) |
| MUCL 28849 | CRISPR-Cas9 | pCRISPRyl; | Lithium-acetate transformation | HR (EasyCloneYALI) | Integration of the LIP2 gene under the control of different promoters (EXP1, pH3, UAS1B8-TMAL(250)), in one or multiple copies, to compare lipase activity. | Identify, characterize and develop strong glycerol-active promoters for use in bioprocesses with *Y. lipolytic*a, including the creation of hybrid promoters and application in the LIP2 gene (extracellular lipase). | <https://doi.org/10.3390/microorganisms11051152> |
| PO1f | CRISPR-Cas9 | pCRISPRyl-AXP | - | HR | Heterologous introduction of the enzymes: TAL, 4CL, and ST Expression of the R3GAT gene  deletion of the EXG1 and BGL2 genes | Polydatin production | <https://doi.org/10.1016/j.biortech.2023.129129> |
| PO1f | CRISPR-Cas9 CRISPR-Cas12a | pHR_A08_Cas9 | Electroporation | NHEJ  HR | Construction of CRISPR-Cas9 and Cas12a libraries: multiple knockouts distributed throughout the genome; large-scale mutagenesis for fitness analysis | Identify genes essential for growth. Detect genes involved in osmotic stress tolerance. | [https://doi.org/10.1038/s42003‑023‑04996‑8](https://doi.org/10.1038/s42003%E2%80%91023%E2%80%9104996%E2%80%918) |
| PO1f | CRISPR-Cas9 | pCRISPRyl | Yeast Transformation Kit (Zymo Research Corporatio) | HR | 14 genes related to tolerance to acetic acid and furfural | Tolerance to lignocellulosic inhibitors, acetic acid and furdural | <https://doi.org/10.1016/j.biortech.2024.130764> |
| PO1f | CRISPR-Cas9 | pINA1269, pINA1312 | Yeast transformation kit (Zymogen Frozen EZ); | Homology-directed recombination (HDR) | Knockouts: ylTYR1, ylTRP2, ylPYK | Increased synthesis of 2-phenylethanol (2-PE) via the shikimate/Ehrlich pathway | <https://doi.org/10.1016/j.biortech.2024.131354> |
| PO1f | CRISPR-Cas9 | pSC012 | Pooled transformation + growth screening (Chen et al. 1997) | NHEJ | Genes for acetate and hydrocarbon tolerance | Rapid tolerance/growth engineering | <https://doi.org/10.1016/j.ymben.2024.09.005> |
| PO1f | CRISPR-Cas9 | pGGDHR | Yeast transformation kit (Zymogen Frozen EZ) | Homology recombination (Golden Gate + HR) | PEX10 knockout; route for arachidonic acid | Production of arachidonic acid | [https://doi.org/10.1007/s10529‑023‑03444‑1](https://doi.org/10.1007/s10529%E2%80%91023%E2%80%9103444%E2%80%911) |
| E004 | CRISPR-Cas9 | pCRISPRPRyl | Lithium-acetate /ssDNA/PEG protocol | HR | Gene editing of the glycolysis pathway (FBP, FBA, TPI), pentose phosphate pathway (ZWF, GND, RPE, RPI, TKL, TAL), redox shuttle (MDH2) and erythritol-related genes (ER10, ER25, ER27) | Enhanced production of Erythritol | <https://doi.org/10.1016/j.biortech.2023.129918> |
| PO1f | CRISPR-Cas9 | peCASyl, eSpCas9 | Plasmid transformation | NHEJ | TRP1 e LIP2 | CRISPR-Cas9 system optimization | <https://doi.org/10.3390/jof10010063> |
| PO1f | CRISPR-Cas9 | Modified pCRISPRyl + donor constructs | Lithium-acetate /ssDNA/PEG protocol | HR | Integration of fsr1/3, FsPPT1; OE of Tgl4, POX2 | Production of 6-MSA and bostrycoidin | <https://doi.org/10.3389/ffunb.2024.1327777> |
| Po1g | CRISPR-Cas9 | pCRISPRyl-P TEF -nCas9-pmCDA1-UGI | Cas9 integrative plasmid | HR | Deletion of genes related to fatty acid degradation (MEF1, PEX10 and FAD2) | Production of ricinoleic acid | <https://doi.org/10.1016/j.ymben.2023.12.002> |
| W29_ST6512 | CRISPR-Cas9 | Cas9 integrative plasmid | EasyCloneYALI Kit | HR | Deletion of ARE1, DGA1, DGA2, LRO1, ICL1, IDH2, IDH2 and IDP, | Efficient synthesis of itaconic acid | <https://doi.org/10.1126/sciadv.adn0414> |
| PO1f | CRISPR-Cas9 | pCRISPRyl | Lithium-acetate /ssDNA/PEG protocol (Chen, Beckerich and Gaillardin, 1997) | HR | Inativation of MET25, YALI0E08536g, YALI0F14047g, YALI0D17402g, CYS3, CYS4, HOM2, HOM3, or MET | Heavy metal remediation | <https://doi.org/10.1016/j.jhazmat.2024.134954> |
| W29 | CRISPR-Cas9 | pSZ_EC_02_ pCfB6371 | EasyCloneYALI Kit | HR | Obtaining a new strain W29Xyl | Growth with xylose as a sole carbon source and and lipid accumulation | <https://doi.org/10.1016/j.biortech.2024.131558> |
| PO1f | CRISPR-Cas9 | pCRISPRyl | IITM Frozen-EZ Yeast Transformation Kit (Zymo Research) | HR | Erythrose reductase (ER10) deletion | Reduce the production of by-products and improve the conversion of glycerol to erythritol | [https://doi.org/10.1007/s12257‑024‑00005‑9](https://doi.org/10.1007/s12257%E2%80%91024%E2%80%9100005%E2%80%919) |
| PO1f | CRISPR-Cas9 | pCRISPRyl | Lithium-acetate /ssDNA/PEG protocol  . | HR | MFE1, PEX10 and POX2 | Increased lipid production | <https://doi.org/10.1016/j.crbiot.2024.100189> |
| PO1f_ XK17 | CRISPR-Cas9 | pCAS1yl | Frozen-EZ Yeast Transformation II kit | HR | knockout of MFE1 (β-oxidation), integration of synthetic pathway genes for docosahexaenoic acid (DHA) | Production of DHA (high-value polyunsaturated fatty acid) | [https://doi.org/10.1007/s10529‑024‑03534‑8](https://doi.org/10.1007/s10529%E2%80%91024%E2%80%9103534%E2%80%918) |
| PO1f | CRISPR-Cas9 | pYJS-Cas9 | Lithium-acetate /ssDNA/PEG protocol | HR | Knockout of genes in the decomposition of erythritol pathway (EYD1, EYK1, EYL1, EYL2 and EUF) | Increased erythritol production | <https://doi.org/10.1016/j.biortech.2024.131035> |
| W29, , YLSL02YLSL01 | CRISPR-Cas9 | CAS9-VPR | Plasmid and casset transformation by lithium-acetate | HR | Overexpression of RAD genes | Construction of a strain specializing in homologous recombination (HR) | <https://doi.org/10.1016/j.tibtech.2025.06.009> |
| PO1f | CRISPR-Cas9 | pCRSPRyl | Plasmid transformation by lithium-acetate | HR | Knockout of genes related to chitin (chs3, chs4, csm1, csm2), hyphae (hoy1, mhy1, snf5, rac1, cdc25) and mannoprotein (pir1, cwp1) | Cell wall engineering to improve extraction efficiency of functional lipids | <https://doi.org/10.1016/j.fbio.2025.106678> |
| PO1f Δku70 | CRISPR-Cas9 | - | Single plasmid transformation using the Zymogen Frozen-EZ II Yeast Transformation Kit (Zymo Research Corporation) | HR | _ | “*de novo*” biosynthesis of δ-tocotrienol | <https://doi.org/10.1016/j.synbio.2025.02.011> |
| YE54 | CRISPR-Cas9 | - | Single plasmid transformation using the Zymogen Frozen-EZ II Yeast Transformation Kit (Zymo Research Corporation) | - | - | *De novo* biosynthesis of dihydroquercetin with enhanced (2S)-eriodictyol | <https://doi.org/10.3389/ffunb.2024.1327777> |
| PO1f | CRISPR-Cas9 | pCas9- POX2 | Single plasmid transformation using the Zymogen Frozen-EZ II Yeast Transformation Kit (Zymo Research Corporation | - | - | Optimizing longifolene production | <https://doi.org/10.1016/j.synbio.2025.01.004> |
